# Supplementary material for: Individual-differences affecting emotion regulation behaviors of injured athletes: A retrospective quantitative study
Source: PLoS One. 2023 Nov 21;18(11):e0294551. doi: 10.1371/journal.pone.0294551 (PMC10662789; doi:10.1371/journal.pone.0294551)
Supplement: Attachment — Submitted filename: Response to Reviewers.docx [file pone.0294551.s002.docx]

**Response to academic editor**

I have responded to all of the reviewers' comments.

The emphasis in this revision was to specifically describe how control variables were handled. In connection with this attempt, I intend to further add information on the attributes of participants, research procedures, and analysis procedures to ensure the transparency of results.

Thank you for your continued support.

**Response to reviewer**

**Reviewer #1**: The research addresses a relevant topic namely the emotional responses people feel when injured. The authors make speculative hypothesis regarding the emotional impact of injury and seek to test these hypotheses among a group of student athletes. There is some merit in the research method that is followed. However, there are some limitations regarding the conceptual framework underpinning the work.

**<Comments>**

The first part is the notion that the emotional response to injury is fixed and not transient. At different stages of an injury people experience different emotions. The research design does not accommodate this.

**<Responses>**

The transformation of emotional responses is well understood by the author, as grief response model and integrated model have been presented. I think you have pointed out that the emotion regulation behavior at each time is different due to the change in emotions. This is a very important comment.

However, this study has a different nuance. Participants were asked to respond by macroscopically looking back on their injuries. The purpose of present study is to quantitatively examine from a macro perspective, "what kind of emotion regulation behavior is predominantly used and what kind of personal variables are related to the background of that emotion regulation behavior".

**<Comments>**

Second limitation is the suggestions that causal relationships are being tested. All data were collected at the same time and therefore the possibility that the direction of relationships goes both ways is plausible.

**<Responses>**

A longitudinal study of injured athletes is appropriate for quantitatively determining the causal relationship. Alternatively, it may be possible to provide an impactful hypothesis after accumulating case studies and qualitative studies. However, the former method has difficulties in research ethics (although there may be researchers who carry out research, it is difficult to obtain cooperating institutions). The latter is not suitable as a method for examining the overall tendency of the injured athlete's response, and remains a strong hypothesis.

The intent of this study is to quantitatively support the viewpoint of avoiding the abnormal aspects such as the prolongation, fixation, and repetition of grief caused by the suppression from the large sample. Therefore, in the present study, the retrospective questionnaire survey method was adopted.

I understand the points made by the reviewers. However, there are many studies that estimate causal relationships from data taken simultaneously in time. In general, in order for this kind of study to be successful, it is necessary to clarify the relationship between variables based on previous studies and theories. In the present study, I intended to discuss it in the introduction.

**<Comments>**

When these two factors are taken into account, it undermines the believability and contribution of the work considerably. It is possible to re interpret the data and treat the study that is exploring relationships. This represents a very different type of study and one that is more suitable in a different journal.

**<Responses>**

I think that the reliability of the causal model was improved by estimating the fit of the model examined by the orthodox method using the goodness-of-fit index by SEM. Of course, I also recognize that there are limits. For this reason, I have stated the future direction at the end of the Chapter of Limitations of the present study **(L420-429)**.

In the future, I would like to believe that complementary discussions will be possible by adding the results of longitudinal qualitative studies to the results of this quantitative study.

**Reviewer #2**: I thank the authors for the work done. Despite being a cross-sectional study, the manuscript is well developed, however, some adjustments are necessary to improve the presentation of the results, therefore I suggest the following:

**<Comments>**

It would be interesting to add the characteristics of the sample: how many female? what sports did they practice? at what level?

**<Responses>**

As shown in **L145-153**, in addition to the sex and age, the competition level and the category of injuries of participants, the competition type is also described. Also, Table 1 is newly added.

**<Comments>**

Please incorporate study design

**<Responses>**

Shown in **L125**.

**<Comments>**

Add the registration number of the ethics committee

**<Responses>**

Shown in **L170**.

**<Comments>**

On line 247 please add the p-value in each variable

**<Responses>**

Shown in **L260** and **261**.

**<Comments>**

Were analyzes carried out distributing the group by sex, age, sport or competitive level? I think it would be important to clarify it.

- The regression models were adjusted for some factor (age, sex, etc.)

- Although the discussion is appropriate, it is necessary to contrast the literature according to the characteristics of the sample (sports practiced, competitive level, etc).

**<Responses>**

Thank you for your plausible comments. The above three comments are related, and I would like to answer them together below.

The present study focused on individual variables and emotion regulation behavior, and examined the relationship between them. Already in the first manuscript, I have conducted correlation analysis, partial correlation analysis, and multiple regression analysis to confirm whether sex, age, sport, competition level, and degree of injury affect the objective variable.

As a result, I have confirmed that the results are not affected even if these variables are not used as control variables.

In the revised manuscript, I decided to concretely show the background of not assuming control variables based on the results of correlation analysis and partial correlation analysis, which are general confirmation procedures **(L268-283 and Table 2 lower part)**. The description of the results of multiple regression analysis assuming control variables is omitted for the following reasons.

Scholars are in a position to encourage and do not encourage the assumption of control variables in multiple regression analysis. The reason for the latter is that the arbitrariness of the researcher is suspected in that the input of variables automatically increases the coefficient of determination (*R^2^*). In the results of multiple regression analysis, no significant path coefficient was confirmed from the assumed control variables, and the coefficient of determination (*R^2^*) of the regression equation was almost unchanged even when the control variables were added. In other words, I would like to emphasize the contribution of the three individual variables (athletic identity, sports ethics commitment, and difficulty describing feelings) to the coefficient of determination (*R^2^*).

Based on the above, in this study, I attempted to focus on the relationship between personal variables and emotional regulation behavior. However, the comments on the possibility of approaching individual differences in response are suggestive, and I would like to leave this as an issue for the future.

**<Comments>**

I think it is possible to add a conclusion

**<Responses>**

Added chapters on conclusions and clinical implications **(L432-437)**

**Reviewer #3**: This is an interesting article which aimed to examining individual variables and variables related to positive reappraisal what should help screen injured athletes that might show abnormal responses caused by suppression.
The theme of the article is relevant because still little is known about relationship between individual variables and emotion regulation behaviors.
Whilst the article is interesting, several points are worth addressing.

**<Comments>**

Abstract:

Line 19 - add measured variables, add information what kind of relationship was evaluated and which statistical methods were used (e.g. correlations, regression).

**<Responses>**

Added to **L19-23**.

**<Comments>**

Introduction:

General comment: use impersonal form permanently (e.g. Following hypotheses were developed ... instead of I developed the following hypotheses....).

**<Responses>**

There were dozens of relevant sentences. I fixed everything.

**<Comments>**

Line 74 - should be relationship instead of correlations (regression was also used in analysis).

**<Responses>**

Corrected according to **L76**.

**<Comments>**

Line 109 - delete heading

**<Responses>**

Deleted.

**<Comments>**

Lines 70-76 - should be combine with established hypotheses at the end of Introduction paragraph

**<Responses>**

The text of **L75-76** was used, and the unnecessary text that followed was deleted.

**<Comments>**

Methods:

Line 131 - exclusion criteria should be presented
above in relation to line 144 - how many participants (men and women) didn't meet each criterion

**<Responses>**

Exclusion criteria are shown in **L131-133**. The number of participants (number of men and women) who did not meet the criteria was listed in **L145-148**.

* I may have misinterpreted the comment. In that case, please point out again.

**<Comments>**

line 143 - questionnaire should be shortly described; was the questionnaire earlier published (citation needed) or it was authors survey (information about validation procedure needed). What kind of injuries were registered? It should be explained.

**<Responses>**

Excuse me. I didn't understand the comment well. I explained the scale included in the questionnaire to the method. Is it different from that information? Please teach me again.

**<Comments>**

Line 194 - Heading: Statistical procedures instead of Analysis method

**<Responses>**

Corrected according to **L205**.

**<Comments>**

Line 197 - Interpretation of Cronbach alpha with internal consistency range (rules of thumb) should be presented.

**<Responses>**

Described in **L208-209**.

**<Comments>**

Results:

Line 239 - Basic descriptive statistics of the variables used in article should be calculated (mean, sd, 95% CI) and presented in first table.

**<Responses>**

Shown in **the left column of Table 2**.

**<Comments>**

Discussion:

Discussion is generally well-written. The summary, however, lacks any specific information as to whether the hypotheses have been rejected or accepted.

**<Responses>**

In the first manuscript, the last sentence of the first paragraph of Discussion stated that the hypothesis was supported, but it was stated at the beginning. In addition, a new paragraph was added at the end of the Results to summarize the analysis results **(L323-332)**, and it was stated that the hypothesis was proved at the end of the paragraph **(L331-332)**. This improved the connection between the results and the discussion, and I think it was possible to concretely show that the hypothesis was tested.
