# Supplementary material for: Individual-differences affecting emotion regulation behaviors of injured athletes: A retrospective quantitative study
Source: PLoS One. 2023 Nov 21;18(11):e0294551. doi: 10.1371/journal.pone.0294551 (PMC10662789; doi:10.1371/journal.pone.0294551)
Supplement: Attachment — Submitted filename: Response to Reviewers.docx [file pone.0294551.s003.docx]

**Response to academic editor**

I apologize for the delay in submitting the revised manuscript.

In this revision, I have incorporated the comments of two reviewers (Reviewer 1 and 2). If the content is still insufficient, please point it out.

Thank you for your continued support.

**Response to reviewer**

**Reviewer #1:** I have read the responses to my comments which we intended to enhance the paper and give the author a window or him to see how the work comes across. In terms of the first response, that a macro perspective was taken. the work should be framed in a macro perspective and the theoretical nature of that approach explained. An especially serious point to make here is the influence of memory on the recall of emotions and how they are regulated, something made difficult when intense emotions are experienced on a regular basis. Many issues here in my view, largely dismissed by the author.

A second major issue is the interpretation of data. The argument that it is a causal analysis when it is a correlational analysis of memories over time but assessed at a single point in time. The effect of current mood on the recall of information could be large in some people and as such ambient mood might explain what information is recalled. Adding alpha coefficients misses the point.

**<Responses>**

I take the reviewer's comments as strict and correct comments. I think that problems such as memory problems that recall bias can occur and contradictions in causally analyzing data obtained at the same time are inherent in the design of this study. It is difficult to completely eliminate this problem, and this research addresses these problems in the following ways.

First, as a countermeasure against recall bias, in the questionnaire survey, as specified in the method, active student athletes were targeted and asked to pick up one injury experience (rehabilitation process) after entering university. Next, at the beginning of the survey, they were asked to give a general review (recollection) of their post-injury rehabilitation process, and were guided to respond to each scale. Although it is difficult to explain the validity of this method using existing theories, a procedure similar to the wash-out period that is conducted in clinical trials was taught at the start of the questionnaire survey. This is a common research procedure in this study design.

In addition, in order to address this bias, athletes who are currently injured, those who were unable to comeback to sports due to injury, or athletes who have returned to competition for less than a week have been excluded from the survey. In addition, as indicated in the procedure for consent to surveys, survey cooperation is not compulsory. Athletes who had difficulty recalling their injuries for various reasons were allowed to stop responding midway. As a result, there were no applicable athletes.

In other words, it can be assumed that the data analyzed in this study were obtained from participants who were able to face and recall their own injury experiences. Errors can occur in any study design. I believe that using large-scale data offsets such problematic effects. Although not perfect.

Next, regarding the direction of causality between variables, no matter how I devised the text of the instructions and questions, I think that the data obtained at the same time will not change. However, individual variables have a trait-level nature. Therefore, I interpret that it is possible to make causal inferences about how the three types of individual variables influence the choice of emotion regulation behavior.

If the above ideas and methods are naive, I think this is the limit of this study. I understand that the design of this study is not rigorous. However, due to the nature of the data dealing with the mind of injured athletes, a quantitative study based on retrospective data was first performed. There are no quantitative studies in this field. The conclusion of this study must not go beyond the realm of speculation, but at least I believe that I have provided a speculation that can be passed on to the next stage of study.

I won't take your comments lightly. Thank you very much.

**Reviewer #2:** I thank the authors for the new version. However, I believe that some points made in the previous review were not addressed, for example indicating the study design and conclusions.

**<Responses>**

I intended to address the above two points in the previous revision. However, I accepted that there was a lack of content, and rewrote everything.

First, the study design was specifically described at the beginning of the method. I also rewrote the conclusion. Please let me know if the content is still insufficient.

Your comment saved me. Thank you very much.

**Reviewer #3:** I have no further comments. All previous recommendations were fulfilled. In my opinion, the article meets the publication requirements of such a respected journal as PlosOne. Congratulations to the authors.

**<Responses>**

Your comment saved me. Thank you very much.
