# Supplementary material for: Individual-differences affecting emotion regulation behaviors of injured athletes: A retrospective quantitative study
Source: PLoS One. 2023 Nov 21;18(11):e0294551. doi: 10.1371/journal.pone.0294551 (PMC10662789; doi:10.1371/journal.pone.0294551)
Supplement: Attachment — Submitted filename: Response to Reviewers.docx [file pone.0294551.s004.docx]

**Response to academic editor**

The revision points were shown in the cover letter. The response to the points pointed out by the editor was shown in the cover letter as a revision point.

Since the comments received from the editor are based on the reviewer's comments, I will provide feedback to the reviewer with the same response as given in the cover letter.

Thank you for your continued support.

**Response to reviewer**

**Reviewer #1**: The issues raised throughout the review process relate to the potential influence of current mood and recall of affective experiences and the use of causal language when the design of the research is correlational. The main response is that there are few studies. Whilst this is agreed, the study does not control for current mood as suggested by the authors in the revision. They needed to explain how this occurs but did not.
 The second is the use of causal terminology - this remains. Sadly, and so the findings are overemphasized.

**<Responses>**

I accept that there is a problem with stating that a 'causal relationship' was revealed in this study design. Certainly, regression analysis does not treat predictive and causal directions the same. Therefore, in this revision, I deleted all the words "causal relationship" and changed it to "relationship".

In addition, in this revised manuscript, I have included in the final chapter as the caveats points to note the "problems regarding the influence of memory bias and psychological state during the response survey" that you pointed out. This is the limitation of this study.

Finally, In the previous manuscript, the " Limitations of the present study " chapter was put first, and the "Conclusion" chapter was later. However, in this revision, the chapters are arranged in reverse. The reason is that similar sentences (sentences about future issues) were repeated at the end of each chapter, and I thought it was better to highlight the caveats points at the end.

That's all.

Thank you very much.
